# Combining Users' Activity Survey and Simulators to Evaluate Human Activity Recognition Systems

**DOI:** 10.3390/s150408192

**Published:** 2015-04-08

**Authors:** Gorka Azkune, Aitor Almeida, Diego López-de-Ipiña, Liming Chen

**Affiliations:** 1 DeustoTech—Deusto Institute of Technology, University of Deusto, Avda Universidades 24, Bilbao 48007, Spain; E-Mails: aitor.almeida@deusto.es (A.A.); dipina@deusto.es (D.L.-I.); 2 School of Computer Science and Informatics, De Montfort University, Leicester, LE19BH, UK; E-Mail:liming.chen@dmu.ac.uk

**Keywords:** evaluation methodology, activity recognition, synthetic dataset generator, activity survey

## Abstract

Evaluating human activity recognition systems usually implies following expensive and time-consuming methodologies, where experiments with humans are run with the consequent ethical and legal issues. We propose a novel evaluation methodology to overcome the enumerated problems, which is based on surveys for users and a synthetic dataset generator tool. Surveys allow capturing how different users perform activities of daily living, while the synthetic dataset generator is used to create properly labelled activity datasets modelled with the information extracted from surveys. Important aspects, such as sensor noise, varying time lapses and user erratic behaviour, can also be simulated using the tool. The proposed methodology is shown to have very important advantages that allow researchers to carry out their work more efficiently. To evaluate the approach, a synthetic dataset generated following the proposed methodology is compared to a real dataset computing the similarity between sensor occurrence frequencies. It is concluded that the similarity between both datasets is more than significant.

## 1. Introduction

Human activity recognition has become a very important research topic, since it is a key technology in applications, such as surveillance-based security [1–3], ambient assisted living [4–6], social robotics [7] and pervasive and mobile computing [8,9]. Even though activity recognition is very diverse in terms of sensing or monitoring approaches and algorithmic choices, evaluation is usually carried out applying the following extensively-used methodology:

1. Choose a target environment and deploy sensors to acquire and process information about human activities.
2. Select a group of persons who can perform the target activities in the prepared environment.
3. Select a dataset labelling system so that the datasets generated by users can be used as a ground truth.
4. Run experiments with users, and label the obtained activity datasets.
5. Use the same datasets to test the activity recognition system and store the labels produced by it.
6. Compare the labels of the activity recognition system with the ground truth using appropriate metrics.

Each of the enumerated steps may vary depending on the activity recognition approach and the available resources. The described methodology, which we refer to as the standard methodology, is the reference for any group working on human activity recognition. The main advantages of the standard methodology are related to the realism, both of the collected data and the behaviour of monitored people. If an activity modelling or recognition approach is validated by the standard methodology, it can be claimed that it should have a similar performance in real-world scenarios.

Nevertheless, there are some problems that make it very difficult to implement the standard methodology. For instance: (i) it is not always possible to own an environment and install sensors and processing systems, due to economic reasons; (ii) running experiments with human beings implies ethical and legal issues that can slow down the research process; and (iii) dataset labelling systems are not perfect, since most of them rely on users' memory or discipline to annotate every activity carried out.

This paper presents a novel evaluation methodology to overcome the enumerated problems. The methodology has been named hybrid, because it combines real users' inputs with simulation tools. The key idea is to circulate surveys among target users with the objective of capturing how they perform certain activities of daily living. Using the information collected by the surveys, individual scripts are prepared, which are then processed by a synthetic dataset generator tool to simulate an arbitrary number of days and to generate perfectly labelled datasets of activities. To get as close as possible to real-world settings, the synthetic dataset generator uses probabilistic sensor noise models and probabilistic time lapses. To enhance the usability of the tool for activity recognition researchers, a detailed methodology has been elaborated, and an intuitive script to model activities and behaviours is provided.

The paper is structured as follows: Section 2 shows the related work. Section 3 describes in detail the proposed methodology. Section 4 outlines the survey designed to capture how different users perform activities of daily living, while Section 5 presents the synthetic dataset generator tool developed to implement the hybrid methodology. Section 7 discusses the advantages and disadvantages of the proposed methodology. Finally, Section 8 presents the conclusions and provides some insights for future work.

## 2. Related Work

Evaluation methodologies for activity recognition systems are usually explained in research papers whose objective is to present contributions related to activity recognition, rather than justifying or validating proposed methods. There are many papers that follow the standard methodology introduced in Section 1, such as [10–12]. Other authors use public datasets provided by research groups, which own pervasive environments and share the collected data. That is the case of [13,14]. The major drawback of such an approach is that those datasets cannot be controlled by researchers and that they may not be appropriate for specific objectives.

A common problem shared by those methodologies refers to dataset labelling methods. Many research papers show experimental methodologies where participants have to manually annotate the activities they are performing (see [5,12,15]). Wren *et al.* [16] show experiments where an expert had to go through raw sensor data to find activities and annotated them. Manual annotation methods are prone to human errors, which result in imperfect ground truth datasets.

There are some alternative methods to manual annotation. For instance, Kasteren and Noulas [11] present a novel method that implies the use of a Bluetooth headset equipped with speakers in order to capture the voice of the participant. While performing an activity, the participant has to name the activity itself. A different approach is presented by Huynh *et al.* [17]. They provide three annotation methods: a mobile phone application, typical manual annotation and another mobile phone application to take pictures regularly and help researchers manually label the activities.

Even though there might be problems when following the standard evaluation methodology, it is clear that it is the best methodology in order to assess the performance of an activity modelling and/or recognition system. However, as Helal *et al.* [18] state in their paper:

Access to meaningful collections of sensory data is one of the major impediments in human activity recognition research. Researchers often need data to evaluate the viability of their models and algorithms. But useful sensory data from real world deployments of pervasive spaces are very scarce. This is due to the significant cost and elaborate groundwork needed to create actual spaces. Additionally, human subjects are not easy to find and recruit. Even in real deployments, human subjects cannot be used extensively to test all scenarios and verify multitudes of theories. Rather, human subjects are used to validate the most basic aspects of the pervasive space and its applications, leaving many questions unanswered and theories unverified.

The solution provided by Helal *et al.* [18] is to develop advanced simulation technologies in order to be able to generate realistic enough synthetic datasets. Indeed, they develop a simulator called Persim, which has been enhanced in the new version Persim-3D [19]. Persim is an event-driven simulator of human activities in pervasive spaces. Persim is capable of capturing elements of space, sensors, behaviours (activities) and their inter-relationships. Persim is becoming a very complete simulator tool for activity recognition in pervasive environments. However, it is still under development, and one of its main limitations is that it does not provide a way to model human behaviour realistically. The authors have already identified this limitation, and they are currently working on programming by demonstration approaches to overcome the problem.

Following those ideas, simulation tools have already been used for activity recognition by other researchers. For example, Okeyo *et al.* [20] use a synthetic data generator tool to simulate time intervals between sensor activations. Their research is focused on sensor data stream segmentation, so the tool generates varying patterns of sensor activations in order to verify their approach. Liao *et al.* [15] combine simulation tools and real data for activity recognition. A more elaborated simulator has been developed by Bruneau *et al.* [21]: DiaSim. The DiaSim simulator executes pervasive computing applications by creating an emulation layer and developing simulation logic using a programming framework. However, it is more focused on simulating applications, such as fire situations, intrusions, and so on, to identify potential conflicts. As a consequence, DiaSim cannot be directly applied to activity recognition.

As can be seen in the literature review, simulation tools can be used for activity recognition, since they provide accurate enough datasets to verify some theories. However, none of the references given above specify a sound methodology to use simulators to evaluate activity recognition approaches. There is no information about how activities should be defined, how different users can be modelled, sensor error models, and so forth, which are key issues when using a simulator. Therefore, there is a lack of a sound methodology that addresses the usage of simulation tools for activity recognition evaluation.

This paper proposes a novel evaluation methodology. The first phase is devoted to capturing user activity and behaviour using surveys, which are subsequently used in the second phase, where a synthetic data generator is used. As the proposed methodology combines surveys for users to capture their behaviour with simulation tools, it is called the hybrid evaluation methodology.

## 3. The Hybrid Evaluation Methodology

The hybrid evaluation methodology has been specially designed for activity recognition systems that assume the dense sensing paradigm introduced by Chen *et al.* [22], where an action of a user interacting with an object is detected through the sensor attached to the object. Even though the methodology itself is not limited to specific scenarios, the implementation presented in this paper works for a single user: single activity scenarios, *i.e.*, only one user is considered, and concurrent or interleaved activities are not taken into account.

The methodology has been named hybrid because it combines real users' inputs and simulation tools. The key idea is to circulate surveys among target users with the objective of capturing how they perform certain activities of daily living. Additionally, users are also requested to describe how their days are in terms of defined activities. For example, a user might make a coffee and brush her teeth in weekdays between 7:00 and 7:30 a.m. Therefore, the aim of those surveys is to model real human behaviour, covering one of the major weaknesses of simulation-based evaluation methodologies. Using the information collected by surveys, individual scripts are prepared, which are then processed by a synthetic dataset generator tool to simulate am arbitrary number of days and to generate perfectly labelled datasets of activities. To get as close as possible to real-world settings, the synthetic dataset generator uses probabilistic sensor noise models and probabilistic time lapses.

**Figure 1:**
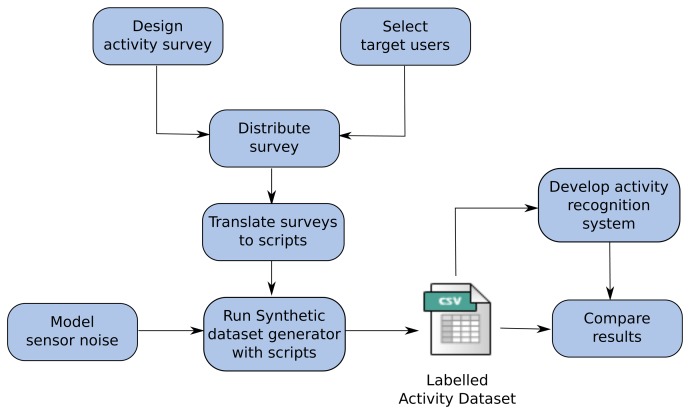
The hybrid evaluation methodology steps depicted in a flowchart.

Based on those constraints and ideas, the proposed hybrid evaluation methodology has the following steps (see Figure 1):

1. Design activity surveys: To capture how users perform activities and to model their behaviour, a proper survey has to be designed. A detailed explanation of how surveys are designed for experimentation can be found in Section 4.
2. Select target users: Depending on the objectives of the research, several user groups can be selected. For example, if the system aims at providing help to elderly people, selecting members of that target group is recommended.
3. Distribute surveys among target users: A suitable way to distribute surveys has to be used, which guarantees users' anonymity. The distribution method can also be influenced by target users. For example, using web-based surveys can be a bad idea if surveys are directed at elderly people, who can be unfamiliar with those technologies. Personal interviews may be a good alternative for those cases.
4. Translate surveys to scripts: Appropriate criteria have to be adopted to translate the answers obtained from surveys to scripts for the synthetic dataset generator, or any other simulator. It is very important not to alter or to lose the information provided by users.
5. Model sensor noise: Sensor noise has to be modelled in order to achieve realistic activity datasets. Real sensors are not perfect, and depending on their technological base, error models have to be provided.
6. Run synthetic dataset generator: Using the scripts obtained from surveys and sensor error models, the synthetic dataset generator is executed. The output of the tool is a labelled activity dataset, which will serve as the ground truth for evaluation.
7. Develop the activity modelling and/or recognition system: Researchers have to develop the activity modelling and/or recognition system in order to be tested. Notice that datasets generated by the synthetic dataset generator can also be used in this step, especially for data-driven approaches.
8. Compare results: Finally, the results obtained by the activity modelling and/or recognition system have to be compared with the ground truth, using appropriate metrics.

## 4. Survey for Activities of Daily Living

One of the main advantages of considering dense sensing-based monitoring scenarios is that activities are described in terms of the objects that have been used to perform that activity. Furthermore, as only sensor activations (and not de-activations) are important for the approach, to model an activity, it is enough to know which objects are used by the user and the order of usage of those objects. This information is easy to obtain in a survey and will be named the activity model.

**Definition 1 (Activity model).** *An activity model is a sequence of objects used by a user to perform an activity. A user might provide several activity models per each defined activity, because the same activity can be performed in several ways. Activity models also provide a typical duration given by the user.*

On the other hand, to model human behaviour appropriately, acquiring activity models is not enough. It is very important to know what activities are performed by a given user on a daily basis, alongside the time slots and time lapses between contiguous activities.

**Definition 2 (Behaviour model).** *A behaviour model is a sequence of activities with associated time slots and time lapses. A user might provide several behaviour models, as every day can be different in terms of performed activities and times.*

The main objective of the survey is to obtain activity and behaviour models from target users. Hence, the survey for activities of daily living has two main parts. The first part is devoted to capturing what activities are performed on different days, *i.e.*, behaviour models (see Definition 2). The second part, on the other hand, asks users about how they perform those activities based on user-object interactions, *i.e.*, activity models. An example of a survey used in some experiments can be found on the web [23].

**Figure 2:**
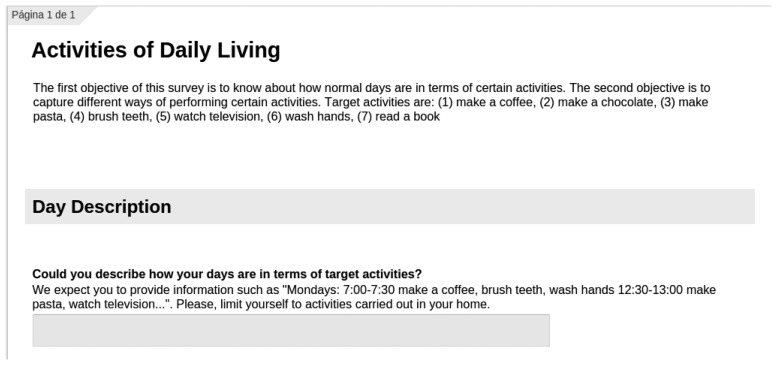
The first part of the survey. A brief introduction can be found where the aim of the survey is explained, continuing with the behaviour model part.

As can be seen in Figure 2, the survey begins with a brief explanation for target users, where the aims of the survey are stated and the target activities are presented. In this case, the target activities are seven: (i) make a coffee; (ii) make a hot chocolate; (iii) make pasta; (iv) brush teeth; (v) watch television; (vi) wash hands; and (vii) read a book. Afterwards, under the heading of “Day Description”, users are asked to describe their week days in terms of activities. They are expected to provide information about time slots and activity sequences performed in those time slots. Users are also asked to provide time relations between two consecutive activities. For example, between 7:00 and 7:30 a.m., a user might make a coffee and ten minutes later might brush her teeth. This first part has been designed to obtain behaviour models for target users.

**Figure 3:**
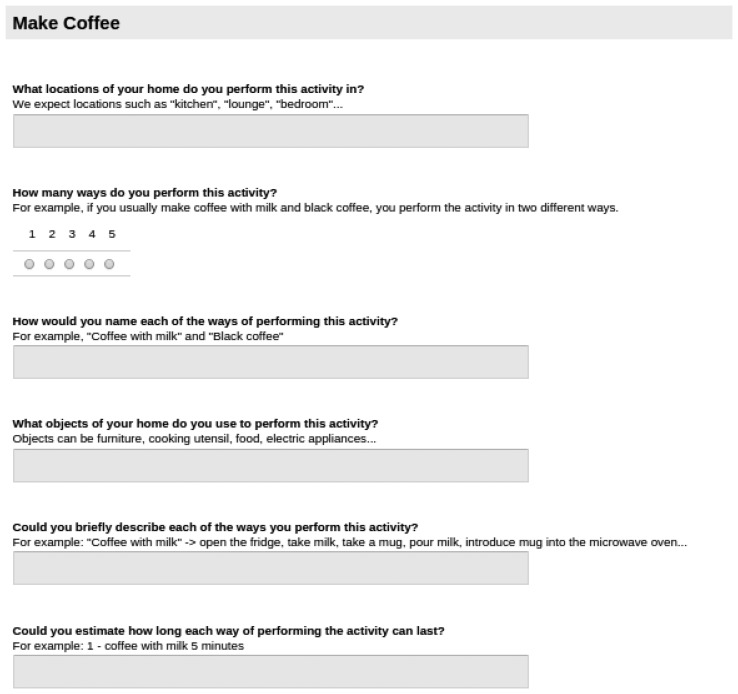
The questions of the survey to capture the activity model of MakeCoffee.

The second part of the survey is longer. Target activities are presented one by one. For each activity, several questions are asked of users, to capture the locations of activities, the ways activities are performed, the objects used for each activity, a description of how those objects are used and typical duration estimations. An example of those questions can be found in Figure 3 for the activity MakeCoffee.

As Figure 3 shows, six questions are asked per activity. The first question is to know where the activity is performed by the user. As stated in the brief explanation under the question, expected locations are home locations, such as the kitchen, lounge, and so forth. Notice that each activity may be performed in several locations; for example, a book can be read in the lounge or in the bedroom.

The second question deals with different ways of performing an activity, *i.e.*, activity variations. Users are asked to provide a variation name for convenience. The next question asks about the objects used to perform the activity. This will serve to model the activity itself, following Definition 1. Afterwards, the most important question for activity modelling comes: a description of how the enumerated objects are used to perform the activity. Descriptions are requested for each activity variation. From those descriptions, object usage order and time lapses will be obtained. Finally, the last question aims at modelling typical durations for the variations of the target activity.

As described in the steps of the hybrid evaluation methodology in Section 3, it is also important to decide the way to circulate the survey and to guarantee user anonymity. In our current experiments, we use the Google Forms [24] service, mainly for three reasons: (i) easy circulation (by e-mail); (ii) users' anonymity is guaranteed; and (iii) simple and centralised answer management is provided.

Summarising, the survey for activities has different questions in order to obtain activity and behaviour models according to their definitions (Definitions 1 and 2). Surveys are circulated among target users using Google Forms, which offers convenient tools to send them by e-mail and collect anonymous answers in a centralised manner. However, depending on the target users, alternative ways can be used.

## 5. Synthetic Dataset Generator

Although the hybrid evaluation methodology could be used in principle with any simulator for human activity recognition, a custom simulation tool has been developed for dense sensing-based monitoring scenarios. Available simulators, such as Persim, do not have the tools to model sensor errors and different variations of activities. Both aspects have been considered important enough to develop a new simulator.

Following the ideas of Okeyo *et al.* [20], instead of developing a simulator that provides visual interaction, like Persim, a synthetic dataset generator has been developed. The tool presented by Okeyo *et al.* does a very good job simulating time relations between sensor activations and activities, so their ideas regarding time management have been borrowed. However, the simulator developed in this paper has more capabilities, allowing researchers to introduce different sensor activation sequences for activities with occurrence probabilities, activity sequences that occur only with a given probability and different ways to model sensor errors.

The synthetic dataset generator tool has been implemented in Python 2.7 [25] The inputs to the synthetic dataset generator are a script called ADLscript, where activity and behaviour models for a specific user are represented, and the context knowledge file, where a list of objects, their locations and attached sensors are provided. For the sensors listed in the context knowledge file, it is very important to provide error models. The considered sensor error modalities are two: positive sensor noise (see Definition 3) and missing noise (see Definition 4).

**Definition 3 (Positive sensor noise).** *A sensor that should not get activated*, i.e., *there is no interaction with the object monitored by the sensor, gets activated because of sensor or monitoring infrastructure errors.*

**Definition 4 (Missing sensor noise).** *A sensor that should get activated*, i.e., *there is an interaction with the object monitored by the sensor, does not get activated because of sensor or monitoring infrastructure errors.*

**Figure 4:**
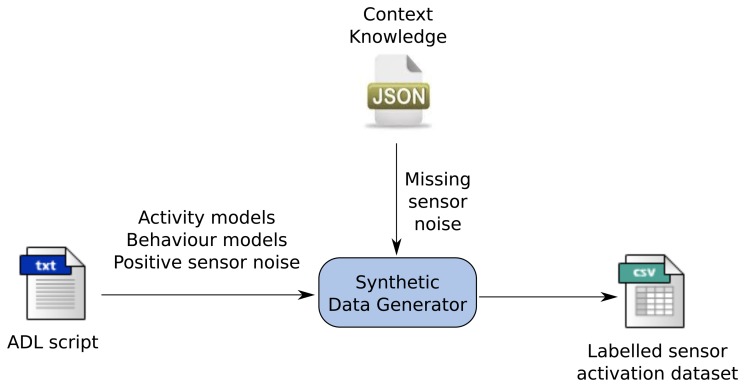
High-level design of the synthetic dataset generator tool.

Figure 4 shows a high-level design for the synthetic dataset generator. As can be seen in the figure, activity and behaviour models and positive sensor noise are represented in the ADL script. On the other hand, missing sensor noise models are obtained from the context knowledge file. Using probabilistic time management tools, the synthetic dataset generator creates a sensor activation dataset, where all sensor activations are properly labelled to use it as ground truth. Sensor activations that are part of an activity are labelled with the activity name. However, sensor activations that appear due to sensor noise are labelled with the special label None.

One of the design decisions was to separate the representation of both sensor error models into two different files. The reason is that missing sensor noise is completely linked to sensor technology and the pervasive infrastructure (wireless receivers, communication and system sampling and conversion mechanisms), whereas positive sensor noise is more related to environmental aspects, such as the distribution of objects and human behaviour. Hence, while missing sensor noise can be considered a property of a sensor, positive sensor noise is more influenced by the inhabitant and the environment. That is why the missing error models are included in the context knowledge file depending on the sensor type and positive error models are represented in the ADL script, which represents a specific user.

To make this decision, the research carried out by Chen *et al.* [26] has been considered. They show some experiments for activity recognition in a smart home environment using the dense sensing activity monitoring approach. Throughout the experiment of 144 activities, a total of 804 user-object interactions were performed. They used the so-called user-object interaction recognition accuracy (UoIR), defined as the system's correctly captured interactions against the total occurred interactions, as the metric to evaluate the reliability and performance of the activity monitoring mechanism. This metric takes into account not only unfired or misread interactions caused by faulty sensors, but also those circumstances caused by the pervasive infrastructure. As such, it is more accurate to reflect the system monitoring performance. Table 1 shows the UoIR for different types of sensors with an overall average UoIR of 96.89%. They conclude that these data prove the monitoring and acquisition mechanism of the system as being very reliable.

However, no positive sensor noise has been identified by Chen *et al.* [26], even though they simulate it in some of their experiments. This is quite reasonable, since the normal state of the sensor represents no interaction. It is very complicated from the technological point of view to change the state of a sensor when no interaction occurs, so it can be concluded that spontaneous sensor activations are very rare. If positive sensor noise is registered, it has to be mainly caused by undesired interactions that actually occur, even though they are not part of the activity. Those undesired interactions can be due to human erratic behaviour (see Definition 5) or interactions among objects caused by their distribution and casual movements.

**Definition 5 (User erratic behaviour).** *This happens when a user interacts with an object, but the object interacted with is not being used to perform the ongoing activity. Consider the case where a user wants to prepare pasta. In order to take the pasta from the shelf, the sugar package has to be removed first. The user will touch the sugar package and thus, a sensor activation will be generated. However, this interaction does not mean that sugar is being used to prepare pasta.*

Given that according to the literature, spontaneous sensor activations are rare and interactions among objects usually occur due to human intervention, it can be concluded that positive sensor noise is mainly caused by user erratic behaviour. Thus, it has been included in the ADL script, rather than in the contextual knowledge file.

### 5.1. ADL Script

The ADL script defines activity models, behaviour models and positive sensor noise for a given user. It is currently implemented as a plain text file, which has its own syntax. A parser function has been implemented to parse the file and to obtain all of the models defined in it.

The first information given in the ADL script refers to the number of days that has to be simulated. A natural number is provided there.

**Figure 5:**
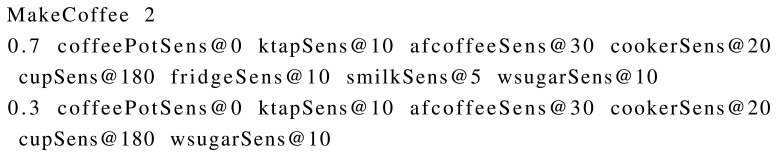
Sensor activation patterns for MakeCoffee activity obtained from a real user via a survey. The activity has two activation patterns with different occurrence probabilities.

The next part of the file is for defining sensor activation patterns for activities. Sensor activation patterns are used to describe how activities are performed in terms of sensor activations and, thus, represent activity models in terms of sensors. An activity can have an arbitrary number of sensor activation patterns, which are specified with an occurrence probability and a sequence of sensor activations with relative time lapses. An example of sensor activation patterns for the activity MakeCoffee can be found in Figure 5.

First of all, the name of the activity is defined. The number that comes after the name specifies the number of sensor activation patterns for that activity. The next line represents the first sensor activation pattern, which begins with an occurrence probability *p* ∈ [0, 1]. Notice that the occurrence probabilities of all sensor activations for a given activity must sum to one. The probability number is followed by a sequence of sensor activations and relative time lapses. The first sensor activation's time has to be zero, indicating that it is the first sensor activation of the activity. The values that come after the “@” symbol represent the time in seconds between the previous sensor activation and the current one. As a consequence, in the example given in Figure 5, *cookerSens@20* means that the sensor activation *cookerSens* occurs 20 s after the *afcoffeeSens* sensor activation. The specific way the synthetic dataset generator treats those time lapses will be explained later, when the simulation loop is described. This representation of sensor activation patterns allows defining different sequences and also the same sequences with different time lapses (and hence, different durations).

Once all activity models are represented using appropriate sensor activation patterns, behaviour models are defined, which represent different days for the user in terms of performed activities (see Definition 2). Two kinds of behaviour models are defined:

1. Sequences: where a time slot is given with a sequence of activities and relative time lapses between two consecutive activities. Sequences are used to define those activity sequences that are always performed by a user in specific days.
2. Alterations: where a probability value is assigned to an activity to be performed in a specific time slot. Alterations represent a different kind of behaviour model. Some users might perform an activity regardless of the week day. For example, a user might watch television in evenings with a certain probability. Some days, the user watches television, but some days does not. It does not depend on the day, but on some other causes (mood, last minute plans, and so forth).

**Figure 6:**
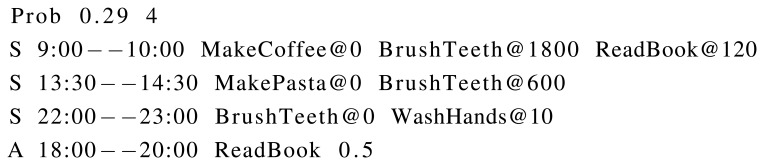
An example of a behaviour model for a specific day, which has an occurrence probability (Prob) of 0.29, and it is composed of three sequences (S) and an alteration (A).

Specific weekdays are not represented in behaviour models: they could be implemented easily, however. Instead of that, the probability of a specific list of sequences and alterations is given. A list of sequences and alterations models a day. Therefore, if such a day model occurs two days in a week, *i.e.*, on weekends, the assigned probability will be 2/7 ≃ 0.29. An example is depicted in Figure 6. A typical day of a user is described, with an occurrence probability of 0.29, since the activity pattern describes a weekend day. In this case, the user reported that (s)he sometimes reads a book in the afternoon. Alterations allow modelling this kind of behaviour.

As happens with sensor activation patterns, the occurrence probabilities of behaviour models must sum to one.

The last part of the script is to define positive sensor noise (see Definition 3). As positive sensor noise is mainly caused by user erratic behaviour, it is very complex to model it accurately. Besides, obtaining those models from user surveys is impossible, since users cannot tell how they interact with objects unpurposely. For those reasons, a simple sensor error model has been adopted that guarantees noise generation independently of ongoing activities. A probability value can be assigned to specific sensors to get activated in an hour interval using a uniform probability distribution. For example, sensor cupSens can be assigned an activation probability of 0.1, which means that each hour, the sensor has a 0.1 probability of getting activated.

### 5.2. Context Knowledge File

**Figure 7:**
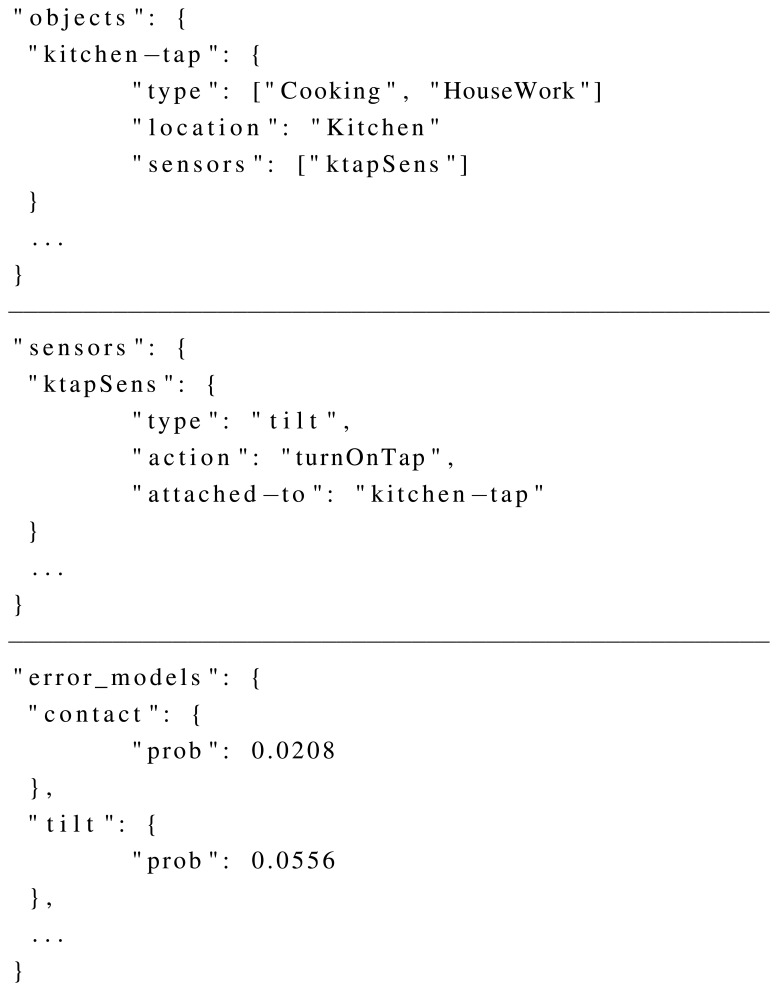
Example of information stored in the context knowledge file, divided into its three main concepts: objects, sensors and error models.

In the current implementation, the context knowledge file is formatted in a JavaScript Object Notation (JSON) [27] file. JSON has been selected because it provides a light-weight knowledge formatting syntax, which is widely supported and used to share information. The context knowledge file is mainly used to represent the objects of the simulated environment and the sensors attached to those objects. Those sensors can be linked to their missing error probabilistic models through the sensor type. Figure 7 shows an example of the information stored in the file. More concretely, several examples are provided for the three main concepts modelled in the file: objects, sensors and error models. In the case of error models, for each sensor type, a missing probability *p* ∈ [0, 1] is provided.

As a consequence, the context knowledge file has the information about objects, sensors and missing sensor noise models. It is mandatory for the synthetic dataset generator to keep the coherence between the sensors used in the ADL script and in the context knowledge file. The tool itself makes sure that such coherence exists. If there is a sensor activation in the ADL script that is not represented in the context knowledge file, the synthetic dataset generator raises an error.

### 5.3. Simulation Loop

Using the ADL script and the context knowledge file, the synthetic dataset generator creates a comma separated value (CSV) file where each sensor activation has an associated timestamp and is labelled with an activity name or with the special label None if it is caused by noise. Additionally, activity start and end times are marked in the dataset.

**Figure 8:**
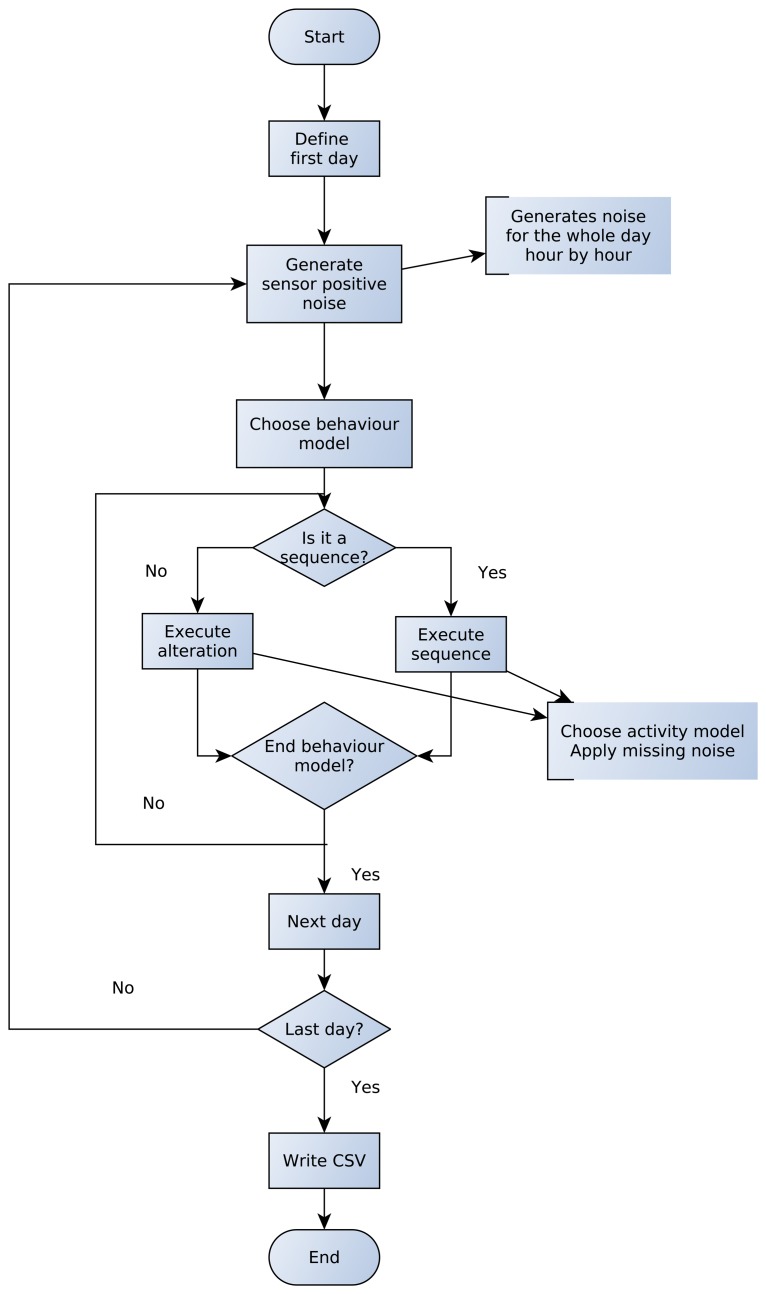
A flowchart of the simulation loop of the synthetic dataset generator.

For the purpose of generating realistic sensor activation datasets, the synthetic dataset generator has a simulation loop that has been represented in a flowchart in Figure 8. First of all, the simulator fixes the first day to start the simulation. In the current implementation the same day the simulator is launched is used as the first day. Afterwards, for the selected day, positive sensor noise is generated. For that purpose, the simulator generates a random number per each sensor with a probability greater than zero. If the sensor has to be activated, the simulator chooses a specific time inside the current hour using a uniform distribution. The process finishes when the 24 h of the day have been treated.

Once positive sensor noise has been generated for the whole day, a behaviour model is chosen, taking into account the probabilities of each model. The behaviour model is a list of sequences and alterations. The first element of the list is taken. If it is a sequence, the activities of the sequence are executed in the specified time slot.

Let us show an example of how a sequence is executed by the simulator. Assume the sequence to be executed is such that:

S 9:00 – 10:00 MakeCoffe@0 WatchTelevision@30 BrushTeeth@1800

In that case, the simulator generates a start time for the sequence in the provided time slot, using a uniform distribution. Such a distribution has been chosen because all times inside the slot should have the same probability, given that the user cannot specify any other information in the ADL survey. Afterwards, it picks the first activity (MakeCoffee) and looks for the sensor activation patterns of that activity. The simulator probabilistically chooses one of the sensor activation patterns of the activity and executes it. While executing the activity itself, two main aspects are taken into account:

1. Time lapses between sensor activations: The time lapses provided in the script are used as the mean values of Gaussian distributions, whose standard deviation is fixed to 25% of the mean by default. This decision has been made because users specify the most common time lapse between consecutive actions. The further the time lapse is from the one specified by users, the lower the probability it has. Thus, a Gaussian distribution models this behaviour properly. Notice that negative values for time lapses are not accepted, since they could change the order of sensor activations. Therefore, the time lapse is generated probabilistically using as a reference the value given in the script. This makes varying and realistic time lapses for consecutive sensor activations.
2. Sensor missing noise: Before generating the sensor activation, its missing probability is consulted in the context knowledge file. The simulator uses the missing probability to decide whether to generate the activation.

Similarly to sensor activations, time lapses between activities are treated through Gaussian distributions. At the end of the process, the whole sequence will be executed, with probabilistically chosen time lapses and sensor missing errors.

To execute an alteration, the simulator uses its occurrence probability. If it has to be executed, the activity is performed in sequences. When the behaviour model is fully executed, *i.e.*, all of the elements of the list have been treated, the simulator generates the next day and repeats the whole process until the last day is reached. When this happens, the generated dataset is written in a CSV file, where properly labelled timestamped sensor activations can be found.

## 6. Evaluation

To evaluate the proposed hybrid evaluation methodology, the idea of Helal *et al.* [18] is followed, *i.e.*, to compare a real activity dataset with a synthetic dataset generated following the methodology described in this paper. For this purpose, the activity dataset published by Kasteren *et al.* [11] has been used. This dataset contains several activities performed by a person in a real pervasive environment. Binary sensors were installed in different objects of the environment, such as in doors, toilet flusher, fridge, and so on.

For the evaluation process, three activities and five days were selected from the complete dataset. More concretely, the selected activities were preparing breakfast, taking a shower and preparing dinner. Those activities were selected because they were executed frequently, following patterns that could be modelled. Additionally, those activities have different durations and quite a lot of variations regarding the object sequences used to perform them. All of those activities are described by the activations of the following sensors: pan cupboard sensor, plate cupboard sensor, cup cupboard sensor, fridge sensor, microwave sensor, hall toilet door sensor, freezer sensor and grocery cupboard sensor.

The typical behaviour model of the monitored person shows that the person usually prepares breakfast in the morning (starting time ranges from 8:00 a.m. to 10:00 a.m.), takes a shower afterwards (10–20 min later) to leave the house. The person comes back in the evening, to prepare dinner, roughly from 6:40 p.m. to 8:30 p.m. Such a behaviour model is provided to the synthetic dataset generator through the ADL script. Objects and sensors are modelled also in the context knowledge file. Using all of the information, five days are simulated, and the generated dataset is compared to the real one.

To compare both datasets, a statistical significance test is applied. The idea is that if both datasets were really generated by the same person and behaviour, the occurrence frequencies of sensors in both datasets should follow a similar distribution. Thus, the time period covered by both datasets (five days in this case) is divided in equally distributed intervals. The interval chosen for the current evaluation is five minutes, because this provides a low-grain view of sensor occurrences, being smaller than typical durations of the described activities. Once the intervals are set, the number of different sensor occurrences are counted for each interval.

**Figure 9:**
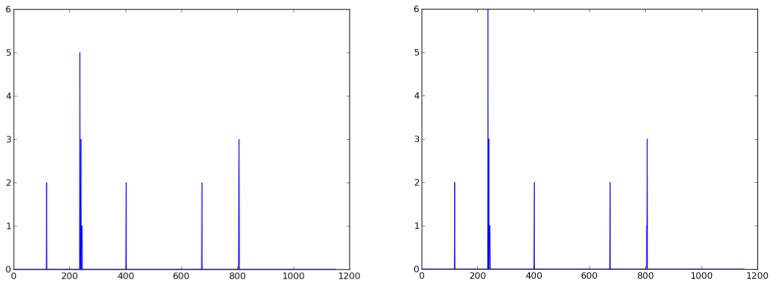
The histograms for the frequency distributions of the fridge sensor: the left graphic is the real dataset, whereas the right one is the simulated dataset.

The information gathered can be represented as histograms of sensor frequencies for each sensor type. For example, those histograms may show that for the interval 9:00–9:05 of a given day, the fridge sensor has been activated twice and the plate cupboard sensor once in the real dataset. It should be checked whether the synthetic dataset shows also similar activation histograms. Figure 9 shows a visual comparison of the real and simulated histograms for the fridge sensor. While the horizontal axis depicts the time intervals, the vertical axis counts the number of sensor activations in that time interval.

The problem of comparing two frequency distributions can be stated as a statistical significance problem, where a synthetic sensor frequency distribution is compared to a real one. Due to the special features of the datasets, Fisher's exact test has been run [28]. Fisher's exact test is similar to the well-known chi-squared test of significance. However, the later cannot be applied in this case, since the frequency values are very low, being zero in the majority of the time intervals. In those situations, Fisher's exact test provides very good results. In this case, the null hypothesis is that both datasets follow a similar sensor frequency distribution, which has been generated by the same behaviour. To discard the null hypothesis, Fisher's test is run, and the *p*-value is calculated (R's implementation of Fisher's test is used in this experiment). Typical significance values for the *p*-value are 0.05 or 0.1. That means that if the *p*-value is smaller than the significance value, the null hypothesis can be discarded. The significance test is run for each sensor, and afterwards, the mean *p*-value is calculated. Results can be seen in Table 2.

For the case of the fridge sensor depicted in Figure 9, the calculated *p*-value is around 0.76. Remember that *p*-value ∈ [0, 1], so it can be interpreted as a similarity measure. Two identical frequency distributions result in *p*-value = 1. As can be seen in Figure 9, both histograms are very similar, but not identical. Notice, for example, that the simulated histogram counts six sensor activations in one of the intervals, while the real one counts five. However, a *p*-value of 0.76 means that both histograms are very similar.

The lowest *p*-value calculated in Table 2 is 0.25 for the hall toilet door sensor. This value has been generated due to a small displacement in the frequency intervals of the sensor activations. The maximum number of occurrences in a five-minute interval is two, so in such a case, a small displacement can lead to a *p*-value of 0.25. Notice though that the *p*-value is still far from the typical hypothesis discarding thresholds, which are 0.1 or 0.05.

However, the most important value is the mean *p*-value, which is around 0.74, as can be seen in Table 2. This means that the null hypothesis cannot be discarded, and thus, the simulated and the real dataset are similar. The graphic depicted in Figure 9, which is highly representative of other sensor activation frequency patterns, suggests that selecting a wider time interval would increase the *p*-value for all of the sensors, whereas decreasing it would also decrease the *p*-value. This has been confirmed by further experiments. However, we believe that for this particular dataset, the five-minute time interval is suitable and meaningful.

## 7. Discussion

The hybrid methodology presented in this paper has several advantages over the standard methodology explained in Section 1:

1. The hybrid methodology is inexpensive and fast: It does not need to acquire or to build any special environment, which can be an important investment.
2. A lot of users' information can be used: As it is based on surveys, it is generally easy to achieve a great number of users for the tests.
3. Ethical and legal issues are much softer: In contrast with the standard methodology, there are no experiments involving humans. The only important point to be considered is the anonymity of users.
4. Datasets can be generated on demand: Using the synthetic dataset generator, an arbitrary number of datasets can be generated as needed.
5. Perfectly labelled datasets can be obtained: The synthetic dataset generator labels all sensor activations according to the given script and sensor error models. As a consequence, the generated dataset is a perfect ground truth.
6. The influence of researchers is minimised: Using surveys, researchers cannot write their own scripts with their biases. Even though researchers are still responsible for writing the scripts, following appropriate survey-script translation criteria, researchers' influence on the datasets is minimised.
7. Any kind of scenarios can be implemented: The synthetic dataset generator allows preparing experiments where no sensor noise exists, where only a specific kind of sensor noise exists or where conditions are as close as possible to realistic settings. The chance to implement all of those varieties of scenarios allows researchers to test their activity recognition systems deeper, since they can see the influence of any factor that they consider relevant.

The results shown in Section 6 prove that realistic datasets can be generated using the hybrid evaluation methodology and the synthetic dataset generator. The mean *p*-value calculated for the significance test where a simulated dataset is compared with a real dataset is high enough to claim that the similarity between both datasets is high. This means that, as far as dense sensing monitoring approaches are considered, the hybrid evaluation methodology can be used to verify complex theories about activity modelling and/or recognition. Indeed, the methodology has already been used in an activity modelling approach by Azkune *et al.* [29].

However, there are some disadvantages, as well. For example, modelling user erratic behaviour is not easy. Although the synthetic dataset generator offers a way to model this kind of interaction, it cannot capture it accurately. Another disadvantage refers to the information provided in surveys. Some users are very precise in their answers, but some are not. Sometimes, important details of activities are omitted by users in their answers; hence, the precise way of performing activities cannot always be captured. Those disadvantages can be coped with specific strategies that might vary depending on the domain. For example, Azkune *et al.* [29] introduce high levels of random positive sensor noise to compensate for the lack of erratic behaviour models. The case of faulty surveys is not an important problem, as long as those surveys can be correctly identified, which is usually the case.

## 8. Conclusions and Future Work

A novel evaluation methodology for activity recognition systems has been presented in this paper. The presented methodology combines the use of surveys for users with simulator tools. Surveys are used to capture human behaviour and how activities are performed. The simulator is used to generate labelled and timestamped synthetic sensor activations according to the behaviour and activity models captured in the surveys. The hybrid methodology is a complete methodology, where it is clearly defined how behaviours and activities have to be modelled and how sensor noise is set in order to use a simulator tool.

To evaluate the performance of the methodology, a sensor activation dataset generated in a real pervasive environment has been compared with a synthetic dataset generated by the simulator described in this paper. The similarity between both datasets has been shown, using a statistical significance test.

It has also been shown that the hybrid methodology has several advantages over the standard methodology used by the community. However, we do not aim to substitute the standard methodology. Our approach can be seen as a good complement to boost research and to let researchers who cannot afford following the standard methodology to make good science. The hybrid methodology is a good methodology to evaluate research works on activity recognition.

For future work, three main areas have been identified: (i) research on more complex and accurate methods to model user erratic behaviour; (ii) adaptation of surveys and synthetic dataset generator to implement the single user-concurrent activities scenario; and (iii) assessing the acceptance and perceived usefulness of the developed tools in the research community, following the criteria identified in [30]. Advances in those three areas would allow simulating more realistic experiments and provide powerful and useful tools to researchers.

The use of positive sensor noise to simulate user erratic behaviour is not enough. Our future approach is to use domain knowledge to simulate such behaviours more accurately. For example, it is common sense to think that there will be more erratic sensor activations during the execution of a specific activity, which are related to objects that are close to the target objects. If a person is preparing a coffee, the erratic sensor activations will usually be related to objects in the kitchen. The plan is to use object location information to generate random activations when an activity is being executed. Further strategies will also be analysed and tested.

For the adaptation of the hybrid methodology to single user-concurrent activities, it is planned to ask in surveys what activities are usually performed concurrently. Only with this information could the synthetic dataset generator try to segment activity models, finding the biggest time gaps and inserting alternate sensor streams in those gaps.

An important way to evaluate the proposed evaluation methodology and the developed tools is to assess the acceptance of and perceived usefulness for the research community. In this paper, the ability to generate realistic datasets for activity recognition has been shown, but the acceptance of the approach has not been addressed. The methodology has already been applied by some researchers, and their acceptance level is high. One of the identified improvement areas is related to the ADL script. Using standard file syntaxes would require less effort from the users, both in the design and maintenance stages. Alternative syntaxes based on JSON are currently being explored to enhance the usability of the synthetic dataset generator.

However, the evaluation for acceptance and usability has to be done in a systematic manner. The idea is to distribute the developed tools among more researchers of the activity recognition community to see whether the approach is useful for them. To assess the acceptance of those researchers, specific surveys will be designed and distributed among those researchers.

## Figures and Tables

**Table 1:** Interaction recognition rate as shown by Chen *et al.* [26].

**Sensor type**	**Total interactions**	**Captured interactions**	**Accuracy (%)**
Contact	624	611	97.92
Tilt	126	119	94.44
Pressure	36	32	88.89
Sound	18	17	94.44

**Table 2:** The *p*-values calculated from the Fisher test for each sensor in both datasets and the mean.

**Sensor**	***p*-value**
Pan cupboard sensor	0.6
Plate cupboard sensor	1
Cup cupboard sensor	0.33
Fridge sensor	0.76
Microwave sensor	1
Hall toilet door sensor	0.25
Freezer sensor	1
Grocery cupboard sensor	1

Mean value	0.74
